# Flipped classroom: Challenges and benefits of using social media in English language teaching and learning

**DOI:** 10.3389/fpsyg.2022.996294

**Published:** 2022-09-23

**Authors:** Shujun Han

**Affiliations:** School of Foreign Languages, Henan University of Animal Husbandry and Economy, Zhengzhou, Henan, China

**Keywords:** flipped classroom, social networks, blended learning, online education, higher education

## Abstract

Due to the emergence of new technologies, reforms in higher education require changes in traditional education. The flipped classroom approach can be a solution to such educational changes to create a student-centered individual learning environment. This approach, which is a type of blended learning, has effectively integrated traditional education and social networks using both environments inside and outside the classroom. The current review is to provide an overview of flipped classroom studies in language teaching contexts. Particularly, the study put emphasis on revealing and addressing the potential benefits and challenges of flipped classroom approach for teaching using social media. It focused on learning environments that students were exposed to the lecture content before the class in a collaborative and interactive learning environment using well-known social media applications. Twenty-five journal publications of flipped classrooms were analyzed in terms of their flipped learning activities, learner achievement, learner attitude, and challenges encountered. The analysis of the selected studies showed that the flipped classroom approach has a positive effect on learning, reducing cognitive load, involvement, accuracy, motivation, attitude, and satisfaction with the course and self-efficacy in higher education, and one of the most important challenges of the flipped classroom is the lack of familiarity and appropriate adaptation of students. With the inversion method, there was an increase in teachers’ workload. According to the analysis of the selected research findings, it is concluded that higher education can effectively use this method.

## Introduction

Today’s students are the citizens, workers, parents, employees, teachers, and leaders of the future (Akçayır and Akçayır, 2018). Student dropout rates show that our education system and the ways in which we prepare students for adulthood need to be reformed (Bergmann and Sams, 2012). Multiple-choice exams continue to assess the knowledge that can be achieved through the lowest levels of learning. Today, traditional lectures in classrooms do not have the necessary effectiveness, because this method causes superficial information to be received that will be forgotten with the passage of time (Hung, 2015; Rajabi, 2015; Mehring, 2016; Webb and Doman, 2019).

According to Mehring (2016), the most pointless thing for students to do is to give long lectures in the classroom and then send them home to do real homework without support. During the last few years, the needs, goals, and performance of learners have changed; they often have easy and quick access to information and prefer to be present in collaborative learning environments with learner-oriented activities (Munir et al., 2018; Mellati et al., 2022). Now the internet and mobile computers are widespread and access to information is easy. It is no longer necessary to keep their information in hand. The world outside of school has changed, while the traditional classroom still stands (Mellati et al., 2013; Aghaei, 2016; Sojayapan and Khlaisang, 2020; Strelan et al., 2020). As Milman (2012) has shown in his research, advanced technologies, the growth of online and available content, and the growth of cognitive science have challenged traditional teaching and learning thinking.

In these situations, one of the methods that can be used to reconstruct the way of teaching and get help from online resources and Internet technology is called flipped classrooms. However, it is necessary to make a fundamental change in the teaching method (Mellati and Khademi, 2014; Aliyyah et al., 2020). In the classroom, in the traditional way, the teacher presents the lesson and moves the class process forward, but in the reverse teaching method, it is up to the students to move the class process forward. Educational technology and activity-based learning are two main elements in the flipped learning model. Both of the above elements affect students’ learning environment in a fundamental way (Bishop and Verleger, 2013).

The reverse learning strategy is one of the learning-teaching strategies that has attracted the attention of international experts in the field of education and upbringing in recent years and can be proposed as a solution to the mentioned problem, during which the usual and current processes of the classroom, especially the lesson delivery section, are shown in the form of an educational film (Mellati and Khademi, 2015; Chen et al., 2018). The recording is given to the students and after watching it and solving the worksheets and exercises, the students come to the classroom with relative preparation (O’Flaherty and Phillips, 2015). In this way, the considerable time that should be allocated for the teaching part is available to the teacher to deal with interactive activities, solving students’ learning difficulties, and generally activities at the higher level of the learning pyramid, which is based on creation, evaluation, and analysis (Mellati and Khademi, 2018; Derakhshan et al., 2020; Lee, 2020). In fact, in this educational strategy, the role of the teacher, student, and educational content is relative to the teaching model. The ritual is reversed and the class time is dedicated to group activities and interpersonal interaction instead of being limited to presenting content (Latorre-Cosculluela et al., 2021). The networks were able to gain a lot of popularity in recent years and this popularity is still increasing, so that they have more than hundreds of millions of users worldwide. These networks have an essential function in various streaming activities with free subscriptions from users. Social networks have been welcomed due to their diverse functions to promote the Internet-based lifestyle (Aguilera-Ruiz et al., 2017; DeLozier and Rhodes, 2017; Lee, 2020). Meanwhile, students and academics are among the main users of educational technologies, including the Internet and social networks (Aliyyah et al., 2020; Collado-Valero et al., 2021). In addition to personal applications, this tool has many uses in education and research, and it can enlarge the level of knowledge and ability of the learners and academics through quick and cheap access to information and scientific resources. The modern intelligent information environment is the result of the emergence and gradual institutionalization of web-based virtual social networks in all human activities of the new century.

While the previous reviews have provided some useful overview over the flipped classroom research, these reviews appear to be inadequate to inform us about the practice of flipped classroom approach in language contexts. Some review studies limited their search only to the higher education context (Almaiah et al., 2020; Wen and Piao, 2020). Some other reviews examined subject disciplines that are usually offered in post-secondary education such as nursing (Mangaroska and Giannakos, 2019; Shi et al., 2020). There are also controversial issue regarding the use of flipped classrooms in the teaching instructions. To the knowledge of the researchers, few literature review study has been done that focuses specifically on the flipped classrooms in language teaching using social media. A systematic review is thus necessary to investigate the implementation of flipped classrooms language teaching field of study. Therefore, the purpose of the current review is to provide an overview of flipped classroom studies in language teaching contexts. Particularly, the study put emphasis on revealing and addressing the potential benefits and challenges of flipped classroom approach for teaching using social media. It focused on learning environments that students were exposed to the lecture content before the class in a collaborative and interactive learning environment using well-known social media applications.

### An overview of flipped classroom studies

Several years ago, two science teachers at Woodland Park High School in Colorado implemented the concept of flipped learning in the classroom. Jonathan Bergman and Aaron Sams decided to use lecture recording software to record introductory science courses and give students the opportunity to watch the lectures as homework (Bergmann and Sams, 2014). Therefore, students had more opportunities to do constructive activities in the classroom. Although the use of the concept of reverse learning in education dates back to 2007, in the early stages of this method, it was limited to teaching online courses through the Internet. In their book Flip Your Classroom, Bergman and Sams describe how they began making videos of their teaching to increase the human, face-to-face interaction students have with each other in the classroom (Bergmann and Sams, 2014; Mellati and Khademi, 2020). Salman Khan officially launched his Khan Academy in 2008, offering step-by-step videos teaching math and science concepts. The idea of using these videos originated from some of his online tutoring sessions with his nieces. He soon realized that by recording these videos, his nieces were able to keep the videos, rewind them, or watch them as many times as needed. Salman Khan began to revise the traditional teaching method to adapt to the 21st century. With the financial support of several philanthropists including Bill Gates, Salman Khan began to build and provide more lessons for a larger class than he had before (Mellati et al., 2018; Andujar and Salaberri-Ramiro, 2021). The flipped class, which is also called by other titles such as flipped class, flipped learning, or reversed classroom, is an educational model in which the traditional way of lecturing and the way students do homework are changed (Almaiah et al., 2020).

### Definition of flipped classroom approach

In the traditional classroom, teachers can only cover cognitive skills up to the level of memorization and understanding with the lecture method. They then send the students home with homework, leaving the core levels of cognitive skills (applying, analyzing, evaluating, and synthesizing) to the student (Wallace, 2014). This is while deep learning involves reaching the same four high levels of knowledge. The student does the assignments alone and maybe with the help of guidebooks and exercise solutions, without analyzing and evaluating, or having an environment to apply and be creative and combine, and a week later to present to the teacher in the classroom (Alavi et al., 2021; Teo et al., 2022).

In the reverse teaching method, however, the teacher’s teaching flow is reversed. First, the teacher prepares the material to be presented in the form of a lecture in the class using educational content production and recording software or from the Internet and provides it to the students (Aguilera-Ruiz et al., 2017). Students see, listen to, and take notes at their own pace. In fact, they acquire the levels of knowledge and understanding in this way and they enter the classroom with the help and guidance of the teacher, interact and discuss in groups with their classmates and face challenging assignments to reach higher levels of cognitive field (Bralić and Divjak, 2018). In other words, the teaching-learning method can be classified into two passive and active forms. In fact, passive learning is done outside the classroom and at home to reach basic levels of cognition, and in the classroom through active learning methods, students acquire high levels of cognitive skills (Cheng et al., 2019; Derakhshan et al., 2021).

The flipped classroom approach is defined as ‘everything that was traditionally done in the classroom is now done outside the classroom and vice versa’ (Asiksoy and Özdamli, 2016). However, simply changing the order of teaching and learning activities is not enough to describe this educational approach. Therefore, Bergman and Sams tried to define the flipped classroom approach systematically (O’Flaherty et al., 2015). As they defined, the flipped classroom approach is a technology-supported educational approach that consists of two components: (1) Individual and direct computer-centered education outside the classroom through video lectures and (2) Group interactive activities inside the classroom (Mellati et al., 2015a; Mangaroska and Giannakos, 2019). Specifically, this definition emphasizes the need to use educational video in learning outside the classroom. However, there are reports of the use of media other than video in the flipped classroom approach, such as the use of presentation files, electronic books, social media, and even paper books (Aydin, 2014).

### Social media in a flipped classroom

One of the most widely used technologies that have been mixed with the lives of many people in the world is the new communication technologies through the Internet. The Internet has been able to play an effective role in various subjects, one of the most prominent of which is the educational aspect (Bakker and Bal, 2010). The Internet has given the teacher many capabilities to create new learning environments, which has brought many advantages. There are two major educational models for using the Internet and the Web teaching network for global learning: Web-based education and Web-based enriched learning. The main distinction between the two models is that Web-based learning uses the Web and Internet technologies as the primary medium for delivery, while Web-based enriched learning uses Internet technologies and resources in the classroom to support Learning and teaching uses (Kim et al., 2014). Web-based training is also called web-based training, web-based learning, or simply e-learning. Web-based education is a form of distance learning that provides education through computers, standard Internet technologies, especially the World Wide Web. Web-based enrichment learning is a classroom-based educational approach that allows learners to use Internet technologies, especially the Web, to access targeted and specific information and human resources in ways that lead to learning (Lindeiner-Stráský et al., 2020). Web-based enriched learning can expand teaching and learning beyond information retrieval to solve problems and lead to knowledge creation. Web-based enriched learning can provide multiple learning contexts for learners and teachers, develop independent learning, and facilitate collaborative communities of learners and teachers (Seaman and Tinti-Kane, 2013; Xie and Derakhshan, 2021). Today, what has caused worldwide attention to education through computers are actually the expansion of the global Internet network and the ease of access to it. It is important to remember that the Internet is a network of networks and connects computer networks around the world to a vast network (Basal, 2015).

Therefore, social networking websites have quickly developed and become well-known in the last 10 years. The extensive use of social networking websites can become optimal as the novel education media to engage learners in social communication (Betihavas et al., 2016). It can be used both for individual contact as well as for teaching goals. Literature shows that the application of social networking platforms has been commonly used in classes, aimed at achieving various learning purposes. Some studies have sought to shed light on the impact of various technologies on the facilitation of language learning. Social networks are sites that allow people to express their beliefs and interact socially with others. Self-expression and social interaction are important contexts for language use (Shtaleva et al., 2021). In the field of education, the positive aspects of social networking sites and their use are being revealed. One of the most important uses of social networks in education is their use in flipped classrooms. Flipped learning can transform traditional teaching methods (Veldthuis et al., 2020). The way of working is that the tests are given to the students online outside the classroom and the assignments that were done at home before are done in the classroom. Flipped learning, as a unique approach, has transformed the role of homework and classroom activities. In the traditional teaching method, students learned new knowledge in the classroom through lectures and practiced them at home. In the reverse learning approach, students learn the material at home through videos and practice the skills in the classroom (Aprianto et al., 2020). Using social networking, the flipped learning model provides an active and interactive learning environment in which the teacher acts as a facilitator and guides students as they apply concepts and actively and creatively engage with the subject matter (Davies et al., 2013).

When the teacher designs and presents a video file appropriate to the subject of the lesson, classroom time is focused on student participation (van Alten et al., 2019). Active learning is done through questions, quizzes, discussion, round table and exploratory activities, art and application of ideas, which play the main role in the flipped classroom model. Nowadays, in classrooms, we can no longer reverse education by reading from books and expressing content in class (Mellati et al., 2015b; Blair et al., 2016). Many students come to class unprepared and teachers do not know how to engage them. Subsequently, it is not enough to say read a chapter of the book and come to class. If the teacher does not have a practical plan to do in class time, all he can do is re-present the same material, effectively implying to students that there was no need to read before class (DeLozier and Rhodes, 2017). For this reason, some teachers at some point decided to prepare video files and send them to their students before class through social media. This enables them to be familiar with interactive methods with the content given to them by the teacher and checks the files as often as they needed, and in return, in the classroom, they gave their teacher the opportunity to present new, experimental and active methods (Attarabeen et al., 2021).

In the traditional education system, the teacher is forced to advance the lesson at an average level, which causes the students who learn quickly to get tired and bored, and those who learn very slowly fall behind. Individual differences and personalizing education is a big challenge, but it is the flipped classroom that allows the student to learn according to their ability (Bredow et al., 2021). It is also a flipped classroom method that allows the student to learn according to his ability. Also, in the flipped classroom method, students are rewarded for real learning. With this method, the student cannot go to the next stage with an unrealistic grade. If he does not learn it completely, he will not be able to go to the next lesson. He should work in such a way that he can show that he has learned the lesson well and completely (Demirel, 2016).

The main problem of direct teaching is that the teacher cannot respond to the different needs of students in learning through lectures. Another problem is how to test students. Different samples of the same test should be given to students so that students cannot give their tests to other students who are taking the same test later (Lai and Hwang, 2016). Because flipped teaching eliminates whole-class lectures, students no longer have to engage in activities in a fixed way. In this method, students do homework, but they do not solve problems alone at home, instead they watch videos. Of course, they can interact while watching the videos. The teacher can ask them to elicit questions from the video, take notes, or share their views on blogs or social networks (Lai et al., 2018).

However, the impact of social networks on teenagers and people at puberty is particularly important because they are a vulnerable group on the one hand and are among the most numerous users of social networks on the other hand (Webb and Doman, 2019). Based on research, 75% of teenagers have user accounts on social networks, of which 68% use Facebook, WhatsApp, and Instagram as their main social networks. Although the use of social networks has an essential role in expanding social communication and learning communication skills, its dangers cannot be ignored (Hung, 2015; Aghaei et al., 2020).

Despite the enormous research on flipped classrooms in the recent decade, little of them has informed research on language learners’ attitude and challenges and benefits of using flipped classroom approach in language teaching and learning. Researchers know surprisingly little about the role of attitude in learning to teach, how flipped classroom approach relate to their students’ achivement. Researchers also know little about how teachers regulate their instructions and use social media, the relationship between teachers and learners in this mode of instruction (Attarabeen et al., 2021; Shtaleva et al., 2021).

### Purpose of review and research questions

The flipped classroom approach is considered as an innovative approach in language teaching and learning (Lai et al., 2018). The purpose of the present review is to understand the impact of flipped classroom approach for teaching using social media, language learners’ achievements, and their attitude toward this new instructional approach. Moreover, the challenges and benefits of employing flipped classroom approach in language teaching and learning field of study were identified. The current review is guided by the following questions:

What is the impact of flipped classroom approach on language learners’ achievements?

What is the language learners’ attitude toward flipped classroom approach?

What are the main challenges and benefits of using flipped classroom approach in language teaching and learning?

## Methods

The search terms used in the present review were as follows: (“flip*” OR “reverse*”) AND (“social” OR “media”) AND (“challenges” OR “benefits”). In this way, the common phases of expressing flipped classroom (e.g., inverted classroom, flipped learning, flipping a class) as well as language teaching could be included. To be included in the present review, the studies must be published in peer-reviewed journals and written in English. The time period of our search was the last decade (January 2010–September 2021). In addition, the studies must be an empirical research reporting an implementation of flipped classrooms in any language context.

By employing the search terms, the researchers found a total of 236 peer-reviewed journal articles. Nevertheless, many articles were removed due to replication across databases. Also, many other articles were found to be irrelevant after reviewing the title and abstract, particularly those were not empirical research or did not involve language teaching and learning. As a result, 25 full-text articles were assessed for eligibility. The researchers contributed to the extraction and categorization of the data. The researchers examined the studies together in order to come to a consensus. The data included major findings, problems encountered, and proposed solutions or preventive strategies to the problems. In particular, the problems identified were analyzed and categorized. The data in each theme were then summarized and synthesized. In following sections, the researchers organized the findings based on the research questions (i.e., the impact on student achievement, student attitude, and the challenges and benefits of using flipped classroom approach for teaching using social media).

### Disadvantages of employing social networks in flipped classrooms

1. **Academic drop:** students who are active in different groups and channels of social networks, their focus on the lesson decreases, therefore, one of the negative consequences of membership and activity in these networks is a decrease in academic motivation and academic drop (Wen and Piao, 2020).
2. **Internet addiction:** social networks by attracting different users, establishing communication, and sharing information create a kind of satisfaction in users; this satisfaction increases the amount of use of social networks and causes students to depend on these networks.
3. **Cyber harassment:** Using social networks can expose students to harassment and inappropriate communication with others and cause them to be abused (Akçayır and Akçayır, 2018).
4. **Being exposed to inappropriate information:** Using social networks can expose learners to issues such as inappropriate, violent content, religious, political, and religious doubts (Basal, 2015).
5. **Violation of privacy:** If students do not check and activate the privacy settings of their user accounts in social networks, they may be abused by cybercriminals (Demirel, 2016).
6. **Creating financial and legal problems:** Failure to observe security and privacy tips in social networks by students can cause financial costs such as hacking bank accounts or publishing confidential information (Lai and Hwang, 2016).

Parents should pay attention to the fact that currently part of the students’ education is done through the virtual space and different educational programs, parents should accompany and pay attention to their children’s activities in the virtual space in order to prevent harm and threats against the students (Lane and Coleman, 2011).

### Learning cycle in flipped learning model

The three basic stages of the reverse learning process are: knowing, interacting, and doing. Teachers should consider these three basic points in the entire educational process (Lo and Hew, 2019). By using this new method, a common characteristic appears in all groups, and that is more dynamics during the classroom. In this case, we are facing students, who are no longer just listening to lectures, but participating in the class and taking full responsibility for their own learning. In this way, they experience different ways of learning: learning by doing, learning by knowing, and learning by sharing with others (Collado-Valero et al., 2021).

In the traditional method, the teacher rarely has the opportunity to involve the students in the activity because he devotes most of his time to lecturing and explaining the lesson content. At best, an active teacher may present the book’s content and images in PowerPoint format in the classroom. In other words, they just read, listen, and see pictures (verbal learning) to progress (Lundin et al., 2018). Probably, the students will eventually reach 30% of learning, but in the reverse learning method, the teacher provides students with verbal and visual learning conditions at home by showing different and attractive educational videos and clips along with recording his speech. Then the students enter the classroom with the preparation of these two learning sources and participate in the activities designed by the teacher in accordance with the subjects of the lesson through group discussion and interaction, and with the activities, they achieve complete learning and use it in different environments (Rienties and Toetenel, 2016).

Today, students are intertwined with technology and the Internet and they are in contact with it almost every day in the classroom and especially outside the classroom and they use it in the best way. As educators, we must use this interest of students in technology and convey educational topics to children through it (Shi et al., 2020). We traditionally do not give students the opportunity to use the most appropriate technology tools to develop their learning outside of the classroom. In the reverse learning method, the teacher also pays special attention to this issue and uses technology as a good teaching tool efficiently. Places instructional videos on websites and blogs for students to view, provides students with a variety of individual activities over the Internet, and communicates with students one-on-one to provide feedback and help (Tang et al., 2020).

### Project-based learning

In the reverse learning method, students examine and analyze a real issue as a project and suddenly come to the conclusion of what solution is appropriate to solve that problem (Lane and Coleman, 2011). Peer learning and teacher feedback were considered as a complementary element to the flipped classroom. These techniques allow teachers to get student feedback the day before class, so the teacher will be able to prepare strategies and activities that the student needs. The teacher can focus on the problems that exist in the way of understanding the contents. The major model emphasizes conceptual content (Wanner and Palmer, 2015).

### Different aspects of learning environment

Review and discussion of selected research findings about the effect of the flipped classroom through four areas of learning, learner, instructor, and technology, which are effective factors in the flipped classroom approach, has been conducted to examine the issue from different aspects related to the learning environment.

#### Learning

Most of the reviewed research confirms that the flipped classroom approach has a positive effect on student learning. One of the important influencing factors is time, so that the students of the flipped class spend more time than the traditional class (Tang et al., 2020). In fact, one of the reasons for the increase in the amount of learning in the flipped class is due to the increase in the amount of work and time rather than the teaching method. Also, in the flipped class, it is possible to receive the educational material at your own pace and repeat it for yourself and discuss the educational material in the group (Andujar and Salaberri-Ramiro, 2021). Of course, the condition of success in the flipped classroom is that the students are prepared to do classroom activities, outside and before the classroom. If this does not happen, the teachers cannot involve them in the classroom activities. The most important way to prepare students is to do homework before class. Doing homework before class instead of doing in the class makes students feel more purposeful in their home activities. Homework is a useful tool for practicing the learned skills, depicting the level of understanding of the course materials, monitoring the learning process of the learner and providing appropriate feedback during the learning process to the learners and the teacher. However, some studies also reported the lack of effect of the flipped classroom approach. Strelan et al. (2020) and Turan and Akdag-Cimen (2020) do not consider the flipped class to be suitable for basic mathematics. The reason for it was shown by Demirel (2016) in his research that the effect of the flipped class is low in subjects where the learner’s basic knowledge is low. Also, Lai and Hwang (2016) did not observe a difference in the learning rate of the flipped class compared to the traditional class. One of the reasons for the lack of difference may be due to the fact that in both studies, the traditional class sometimes turned into flipped class conditions and the students engaged in group activities. In fact, the working method in the class of the control group was the same as that of the experimental group, which had class discussions and work groups which is the reason why the research results did not show a significant difference (Almaiah et al., 2020; Sojayapan and Khlaisang, 2020).

Experts and researchers in different decades believe that interaction is the key element in learning and satisfaction in online and virtual education courses. Usually, the amount and type of interaction in face-to-face classes between students and professors is a challenge. Many of the researches reviewed reported the relationship between the flipped classroom approach and students’ learning by increasing the interaction between themselves and their professors (Wanner and Palmer, 2015; Munir et al., 2018; Almaiah et al., 2020; Sojayapan and Khlaisang, 2020). Another reason is the effect of the flipped classroom on feedback learning. The flipped classroom approach can help to strengthen feedback before, during, and after the class. Feedback helps the learner to reflect on the information provided and their own knowledge, so as to facilitate the correction of misconceptions and the filling of gaps in their knowledge. Also, feedback can be used to measure students’ understanding of learning materials, self-assessment tests, class exercises, and group projects (Andujar and Salaberri-Ramiro, 2021). Clear, purposeful, meaningful, and consistent with the learner’s previous knowledge, specifying a logical relationship is one of the characteristics of effective feedback. Providing feedback using digital technologies adds to the richness of feedback due to the ease of interaction and the possibility of sending comments in each assessment by others. Providing immediate feedback about students’ understanding of online educational materials and evaluating students’ readiness to interact and participate in flipped class activities is achieved by pre-class tests (DeLozier and Rhodes, 2017; Aghaei et al., 2020).

#### Learners

The research study showed that the effect of the flipped classroom approach is not the same for all types of learners, so the characteristics of the audience should be analyzed before starting the course. For example, Basal (2015) has suggested to use the flipped approach in groups with little basic knowledge of the subject, because the level of interaction and involvement in the class will lead to the initial improvement of their situation and those who have less skills in the subject will benefit more (Betihavas et al., 2016). According to Lundin et al. (2018), it was found that academically strong students have no difference in the type of educational model for their progress, but academically weak students in the reverse class approach make significant progress compared to the class. In addition, the flipped class approach is suitable for students with characteristics of interest in open and flexible time, working independently, and feeling in control of learning by themselves (Tang et al., 2020).

#### Instructors

In the flipped classroom approach, the teacher’s role changes from a mere transmitter of information to a guide of learning activities. One of the most important challenges of implementing the flipped classroom approach is its confrontation with the teacher and the traditional classroom. Despite the change of the teacher’s role from instructor to facilitator in the face-to-face classroom environment, the teacher still plays a fundamental role in the effective or ineffective use of technology in this approach (Rienties and Toetenel, 2016). Experts emphasize that if teachers do not experience the pattern of using technology in their classes, it will not be possible to train a new generation of teachers who can use the new tools of information and communication technologies effectively in their learning (Shi et al., 2020). Therefore, it is necessary to pay attention to the capabilities of the trainer in holding a flipped class, because it requires higher skills than the traditional one (Tang et al., 2020). Moreover, instructors should identify students who could not do extracurricular activities or who are facing problems and intervene to solve the problem. One of the reported challenges related to instructors in flipped classes is increasing their workload in producing content and videos for students to use before class (Mangaroska and Giannakos, 2019).

#### Technology

One of the key areas in the flipped class approach is the employed technologies, especially in the two subjects of content production and presentation before the face-to-face class. In content production, the research review showed that the most used media is the educational video, which is actually a video of the teacher’s lecture and teaching (O’Flaherty et al., 2015). To create a more effective flipped learning model, short and meaningful videos should be prepared. Using various methods of video production and combining it with other media can increase the attractiveness and interest of the student to watch the video. In any case, it is possible for the learners to control the video, and they can keep the video or watch it again according to their learning speed (Lindeiner-Stráský et al., 2020).

To investigate the flipped classroom approach fully, by reviewing all the selected researches, the researchers determined the following opportunities and challenges.

### Implications and advantages of using flipped classrooms for teaching through social media

In flipped learning, the main and challenging issue is finding appropriate learning activities, projects, and assignments that require thinking skills. Of course, this case can be considered one of the strengths of this method, and it involves the teacher in the design of activities and practical subjects of classroom learning. The important thing is that in this method thinking about learning results is done instead of thinking about learning content.

#### Development of interaction between teacher and student

Proponents of the flipped learning method believe that this method leads to more interaction between the teacher and the student. For example, Lane and Coleman (2011) acknowledged that when teachers do not tend to stand in front of the class and speak to students, they can move around the classroom and interact with each of their students, in which case, they are more likely to understand students better and to their emotional and learning needs respond. Studies have shown that having teachers who recognize and respond to learners’ social and emotional needs is at least beneficial for learners’ academic development, especially for at-risk students.

#### Opportunity for real-time feedback

Proponents of flipped learning argue that increased interaction between teacher and student gives the teacher more opportunity to provide timely feedback. For example, a case study conducted by the Kim et al. (2014) found that during a five-week summer course where students received instruction through the Khan Academy site with direct teacher support, the teacher spent more time on learning than in a traditional classroom. Therefore, teachers could give the learners more feedback and immediately correct their misunderstandings.

#### Learning at individual pace

Putting teaching on the Internet enables students to learn at their own pace and according to their own needs. According to the research conducted by Sojayapan and Khlaisang (2020) on 800 samples, this learning speed has been one of the most influential factors in the amount of learning interventions. Teachers argue that flipped learning can increasingly enhance a teacher’s ability to provide differentiated instruction. This leads to students learning at their own pace in the classroom.

#### Meaningful assignments

Another advantage of flipped learning is that children complete assignments in the classroom in front of the teacher’s watchful eyes (Seaman and Tinti-Kane, 2013). Alavi et al. (2021) found that providing opportunities for students to practice their skills in class and teacher corrective feedback was nearly four times more effective than homework, where the teacher had less opportunity to guide students during homework.

#### Formative and flexible assessment

Another advantage is that in this way teachers do not leave students alone with homework. Everything is done in the classroom. After a student takes a test or completes a project, the teacher gives them immediate feedback.

#### Changing class management

In this method, the teachers’ role changes and instead of standing in front of the class and controlling the class and being in the center of attention, they guide in small groups, accompany individuals, coordinate activities, and solve their problems.

### Disadvantages of using flipped classrooms for teaching through social media

Flipped learning is a method that helps teachers prioritize active learning during class time by providing some course material for students to observe and study at home or outside of class. However, this educational strategy, like other methods, has challenges that we can better solve by identifying them.

#### Flipped learning is a new approach

The implementation of any new method faces various problems in the first days. Regarding reverse learning, one of the most important challenges is the newness of this method and the unfamiliarity of students with this method and its goals. They may not understand the method and logic of the new classroom, and most importantly, they are not used to learning outside the classroom. The solution to this challenge is that before implementing this method, talk to the students about it so that they experience less confusion in the first days. Explain the purpose of reverse learning to them and assure them that you are not going to leave them alone. Also, assure them that if they have problems understanding the lesson content at home, there is no problem and you are going to solve this problem in class as a group.

#### Active communication in educational videos

Usually, in the reverse learning method, video content is not presented live in an online class. Rather, pre-recorded video educational content is used. If these videos and other types of educational content are not attractive enough and cannot attract the attention of students, reverse learning will not work well. Also, teachers should note that when recording video educational content, they should teach as if the students are in front of them right now and teach in an active manner. In this method, also try to divide the extensive educational contents into short parts, for example 6 min, so that they are not heavy and it is possible to create a sense of some kind of interaction.

#### Lots of assignments from reverse learning challenges

You should never allow students’ workload in flipped learning to exceed the workload in a traditional classroom. In fact, this time should be the same as the students used to do their homework at home. Another important point is that students spend more time watching a video than it actually does because they are constantly pausing and rewinding the video to better understand it. Therefore, each video should not be more than 20 min or at most 30 min.

#### The need for communication outside the classroom with educational coaches

Some students complain about not being able to ask questions to the teacher during recess before class. In fact, if you expect students to be able to use the understanding of the content outside the classroom for the time in the classroom, you should be able to provide them with the best facilities for learning outside the classroom. One of these facilities is increasing communication outside the classroom. This work is provided by creating online groups and forums or using online text, audio or video chat facilities using integrated educational software or even social networks.

#### The overwhelming work for teachers is one of the important challenges of reverse learning

Although there is a large number of pre-existing educational videos on the Internet, some teachers complain that they are not easy to find. Sometimes, these videos or audio educational content or in the form of photos, PDFs and infographics do not fully match the content desired by the teachers for education. On the other hand, the recording of video files by the teachers themselves is a time-consuming and costly task, and in fact, it is not among the duties of the teachers, unless the appropriate time and budget are considered for this task.

The collection of these cases can be summed up in the phrase “exhausting work for teachers.” There are several solutions for this situation: including the involvement of managers and considering the appropriate budget for this method and creating or purchasing suitable educational content and another solution is to provide or create the necessary content not necessarily all at once, but gradually.

## Discussion

The present review overall suggests that the students in language flipped classrooms would have a better achievement, or at least performed equally as in traditional classrooms. This finding was similar to the conclusion of some previous reviews of flipped classroom research in higher education (Betihavas et al., 2016; Aguilera-Ruiz et al., 2017). Unlike some higher education contexts such as Hung’s (2015) review study on chemistry flipped classrooms, the present review cannot draw an overwhelming agreement that students liked the approach language teaching. While student attitude toward flipped classroom approach was generally positive, some studies reported that a few students preferred traditional teaching approach because of the inability to ask questions during video lectures and students being accustomed to traditional instruction (Kim et al., 2014). In particular, Lai and Hwang (2016) found that their students generally reacted negatively toward the change of instructional approach. Meanwhile, the student satisfaction in their flipped classroom was thus significantly lower than that in its traditional counterpart (Lee, 2020).

Flipped classrooms challenges in language flipped classrooms were similar to higher education (Lo and Hew, 2019). First, flipped classroom approach requires a high initial cost particularly regarding the production of instructional videos (Rienties and Toetenel, 2016). Second, teachers should be sufficiently trained in using flipped classroom approach in order to put this approach into full use (Mellati and Khademi, 2018). When compared with higher education, more operational challenges were identified in the contexts of language teaching. Similar to what Shtaleva et al. (2021) found, a few students in language flipped classrooms also suffered from limited Internet access. They may also encounter technical problems and require supports from schools when operating their flipped course (Turan and Akdag-Cimen, 2020).

## Conclusion

This research was a review to investigate the use of the flipped classroom approach in higher education. Reviewing the conducted research helped to better understand the capabilities and weaknesses of using the flipped classroom approach to teaching and learning in higher education. Moreover, the review and analysis carried out on the research conducted in this field can be used as a guide for further research. Furthermore, the findings of this research highlighted the different effects of flipped classroom implementation and blended learning on learning performance. Designing a flipped classroom makes a difference in terms of improving student results with a larger effect size compared to traditional education and electronic learning. Students can spend enough time watching the lectures, enough preparation before participating in the face-to-face meeting where they are active in solving problems based on guided questions. These students receive immediate feedback from the instructor based on guided questions. In fact, the flipped class is an effective combination of online and traditional education for proper use of class time and outside of class (Aghaei et al., 2020).

By designing and implementing a quality curriculum based on the reverse learning approach, we can provide many opportunities for students and teachers to learn and teach. This approach can give students a freedom in learning. Learning in this method is formed more deeply so that it enables students to achieve high level learning. Classrooms also change from a passive and static state to an active and collaborative environment. Interaction and cooperation between teacher and student and students with each other is created and classroom time is used in the best way. Evaluation methods also change from the closed and one-dimensional mode of examination and standardized tests to the real evaluation of the activities and overall results of real learning. Real, timely, and effective feedback is provided. Meaningful assignments are designed. As a result, students become independent and responsible learners who will continue to learn after graduation and will use their learning in the best way in different situations.

Since this method is becoming increasingly widespread in universities and schools, this study is useful for researchers and educators in various fields. In addition, the teachers who are interested in the students’ involvement, the reverse class approach is suggested because these classes bring back the interest in the activity to the class with their active approach. It is suggested that the textbooks be redesigned in accordance with the flipped class, in such a way that more of the content is assigned to homework.

## Further studies

It is suggested to conduct other studies with the criteria that caused the research to be excluded in this research. It is suggested that other researchers investigate the effectiveness of this method on the variables of learning styles, cognitive and metacognitive strategies, and dimensions of self-regulated learning. It is also suggested to check the effectiveness of this method in different environments and learners with different levels (beginner to advanced). Attention should be paid to the gender of the audience, the year of education of the learners, the level of ability of the learners in asking questions, the structure of the class, the way of dividing people in the classes and the class discussion.

## Author contributions

The author confirms being the sole contributor of this work and has approved it for publication.

## Funding

This study was supported by Humanistic and Social Science Youth Fund Project of Ministry of Education, “Research on Grammaticalization and Cognitive Motivation of Chinese and English Verbal Measurement Constructions” (grant no. 20YJC740013), Cultural Revitalization Project of Henan Province, “Research on the Transmission Path of Henan Cultural Symbols from the Perspective of Smart Media” (grant no. 2022XWH112), 2021 Education and Teaching Reform Research Project of Henan University of Animal Husbandry and Economy “Construction and Practice of Interactive Teaching Model Based on Smart Classroom” (grant no. 2021-XJLX-034), and by Doctoral Research Start-up Fund Project of Henan University of Animal Husbandry and Economy (grant no. 2020HNUAHEDF047).

## Conflict of interest

The author declares that the research was conducted in the absence of any commercial or financial relationships that could be construed as a potential conflict of interest.

## Publisher’s note

All claims expressed in this article are solely those of the authors and do not necessarily represent those of their affiliated organizations, or those of the publisher, the editors and the reviewers. Any product that may be evaluated in this article, or claim that may be made by its manufacturer, is not guaranteed or endorsed by the publisher.
